# Sleep Quality in Medical Students; the Impact of Over-Use of Mobile CellPhone and Social Networks

**Published:** 2016-03-27

**Authors:** Abolfazl Mohammadbeigi, Rozita Absari, Farzaneh Valizadeh, Mohammadreza Saadati, Soroush Sharifimoghadam, Ali Ahmadi, Mohsen Mokhtari, Hossein Ansari

**Affiliations:** ^a^ Department of Epidemiology, School of Health, Health Policy and Promotion Research Center, Qom University Of Medical Sciences, Qom, Iran; ^b^ Student Research Committee, Qom University of Medical Sciences, Qom, Iran; ^c^ General Practitioner, Genetic Counselor, Deputy of Health, Mazandaran University of Medical Sciences, Babolsar, Iran; ^d^ Modeling in Health Research Center, Department of Epidemiology, School of Health, Shahrekord University of Medical Sciences, Shahrekord, Iran; ^e^ MSc of Epidemiology, Health Vic Chancellor, Arak University of Medical Sciences, Arakm, Iran; ^f^ Health Promotion Research Center, Department of Epidemiology and Biostatistics, Zahedan University of Medical Sciences, Zahedan, Iran

**Keywords:** Sleep, Social Networks, Internet Addiction, Cell-Phone Over-Use, Mobile Addiction, Students

## Abstract

**Background:** Poor sleep quality is closely associated with lifestyle habits including use of mobile cell-phones. This study aimed to identify the relationship between sleep quality due to abuse in mobile cell-phones and engagement in social networks.

**Methods:** This cross-sectional study was conducted on 380 undergraduate students selected by proportional stratified sampling in Qom, Iran in 2015. Data were collected by two statndard questionnaire including Cell-Phone Over-Use Scale (COS) and Pittsburgh sleep quality questionnaire beside the status of usage in cell-phone social networks. T-test, chi-square, Pearson correlation coefficient and multivariate logistic regression were used in data analysis.

**Results:** The mean age of participants was 21.8 ±3.2 yr, 69.1% were female, and 11.7% were married. The mean of COS and sleep quality scores were 48.18 ±17.5 and 5.38 ±2.31, respectively. The prevalence of over-use of cell phone was 10.7% (CI 0.95; 8.8%, 12.6%) and the prevalence of poor sleep quality was 61.7% (CI 0.95; 57.1%, 66.3%). The mean of all aspects and total score of sleep quality showed a direct significant association by cell-phone addiction score except sleep duration score that was inversely. Based on multivariate analysis affected to cell-phone addiction, being male gender and studying in general physician level are the most important predictors of poor sleep quality.

**Conclusions:** Over use of internet and social networks via smart phones is related to poor sleep quality and quantity. Predefined sport programs, educational, cultural, and interesting entertainment are the essential needs for all medical students. These interventions are more important especially for male students who have longer educational.

## Introduction

Currently, the mobile cell-phones changed as a common tool in the individual’s daily life because people can connect to the virtual networks constantly via internet and the mobile cell-phones not only a talk tool^1^.

The prevalence of Iranian mobile phone and internet usage in Iran increased to 85% and 35%, respectively. Moreover, 22% of Iranian users and 58% of teenagers connect to the internet by their smartphones or tablets^2^. Installing social networks applications besides login to internet social networks by cell-phones caused to an addictive behavior in mobile phone users. More than 500 million people were active members of Facebook in 2011, while according to statistics portal site, Facebook users at the beginning of 2015, was about 1.44 billion. Moreover, Viber and WhatsApp users estimated about 573 and 800 million, respectively and 55% to 82% of adolescents and adults use social networks, regularly.^3^ Social networks enabled interactions between friends and family members via virtual communities based on common interests^3,4^. Social networks can have negative effects, such as anxiety, depression, dependence, and addiction^5-7^,. Besides, it can disturb the sleep quality^8,9^ due to the adverse effects of electromagnetic field emitted by mobile phones and reduced melatonin production^10,11^.

Mobile phone addiction or internet addiction is defined as excessive behavior in using technology tools such as smart phones, android applications or its enteritainments^12^. In addition, people suffer from internet addiction, like those addicted to drugs and alcohol, are faced to excessive problems such as academic problems, social and occupation^13,14^. However, in view of psychologists and sociologists, addictive use of the internet is identifying as behavior problem that affect the sleep quality ^8,9,13,15^. Good quality sleep is essential for health and life quality in all people and is related to several factors including environmental factors, social life, general health status, and stress^1,16^. Todays, mobile phone is one of the important environmental factors that can affect the sleep quality if overused^1,10^. Moreover, the poor sleep quality is related to increase risk of physical and mental disorders and closely associated with lifestyle habits including excessive use of mobile cell-phones^10^. In addition, the effect of sleep quantity and quality on non-communicable diseases such as diabetes is showed^9,17^. However, the medical students experience remarkable stress during long period of study and are prone to sleep deprivation.

This study aimed to identify the relationship between sleep qualities due to overuse of mobile cell-phones and engagement in social networks.

## Methods

This cross sectional study was conducted in a random sample of Qom University of Medical Sciences, Qom, Iran including 380 undergraduate students studying in term 2 or higher in January 2015. Proportional stratified sampling method was used in selecting of participated students and each college defined as strata.

Informed consent was taken from all participants and the study protocol was approved by Ethics Committee of Qom University of Medical Science.

Data were collected by two statndard questionnaire including Cell-Phone Over-Use Scale (COS) and Pittsburgh sleep quality questionnaire. In addition, another questionnaire was used to gather the demographic characteristics and the status of use from mobile cell-phone’s social networks.

### Cell-Phone Over-Use Scale (COS)

The COS was used for mobile cell-phone addiction data^18^ and validated by Iranian researchers^19,20^ and passed the validity tests as well as reliability tests by more than 90%. This questionnaire including 17 items and each item scoring between 1 and 5 for each item in a Likert scale.

After computing the mobile cell-phone addiction score, subjects were categorized in three levels as higher 75 as over-use, 25 to 75 as normal and lower 25 as lower normal^18,20^.

### Pittsburgh sleep quality questionnaire

Pittsburgh sleep quality questionnaire^21^ was used as a standard tool to assess the sleep quality and quantity in students. The Persian version of this questionnaire is validated in some studies in Iranian people^22,23^. Nineteen individual items generate seven “component” scores including subjective sleep quality, sleep latency, sleep duration, habitual sleep efficiency, sleep disturbances, use of sleeping medication, and daytime dysfunction. The sum of scores for these seven components yields one global score as sleep quality score. In each component, the scores varied from 0 to 3 and the total score of questionnaire varied since 0 to 21 and higher scores indicating the worse sleep quality^21^. For each components 0 indicates that no sleep difficulty, 1; mild difficulty, 2 sever difficulty and 3 very sever difficulty in sleep. The total score of sleep quality was categorized based on the lower or higher 5 and labeled as normal and poor, respectively ^21^.

### Statistical analysis

After data gathering and calculation of mobile cell-phone addiction and sleep quality scores for each study subjects, data were analyzed in SPSS software (Chicago, IL, USA). Descriptive statistics including percent mean and standard deviation were used for central and deviation indices. T-test and analysis of variance were used for comparing the sleep quality score between participants based on demographic characteristics (gender, marital status, residency place, educational term) and who used/not used different type of social networks. Pearson correlation coefficient was used for assessing the correlation between cell-phone addiction score with sleep quality score and sleep quality components’ score. Multivariate logistic regression by backward stepwise was method used for adjusting the covariates effect. Goodness of fitness was checked by Likelihoods and Hosmer-lemshow statistics and significant level was considered by 0.05 error.

## Results

The mean age of participants was 21.8 ±3.2 yr, 69.5% were female, and 11.7% were married. In addition, 49.6% were resident in university dormitories. The response rate was 95.5% (363/380). The mean cell-phone addiction score and sleep quality score was 48.18 ±17.5 and 5.38 ±2.31, respectively. The prevalence of over-use of cell-phone estimated was 10.7% (CI 0.95; 8.8%, 12.6%) and the prevalence of poor sleep quality was 61.7% (CI 0.95; 57.1%, 66.3%). 45.6% of study subjects reported that used more than two hours from web, daily. Table 1 shows the demographic and prevalence of usage from social network in mobile cell-phones. Accordingly, more than 58% of students use Viber and WhatsApp applications in smart cell-phones.

**Table 1:** Demographic and prevalence of usage from social network of cell-phone

**Variables**	**Number**	**Percent**
Age group (yr)		
<19	66	18.6
20-21	148	41.7
22-23	87	24.5
≥24	54	15.2
Educational semester		
≤3	148	48.7
4-6	103	33.9
≥7	53	17.4
Gender		
Male	110	30.5
Female	251	69.5
Educational level		
Bachelor	236	65.4
General practitioner	125	34.6
Marital status		
Single	318	88.3
Married	45	12.7
Living		
In dormitories	173	49.6
With family	188	52.4
SIM Card		
One	133	36.9
Over one	228	63.1
Type of social network		
Viber	211	58.1
WhatsApp	221	60.9
Tango	73	20.1
Line	129	35.5
Instagram	101	27.8
Others	62	17.1

Using Viber, WhatsApp, Line and Tango were significantly related to poor sleep quality (Table 2). Moreover, living in university dormitories was related to higher score that indicated poor sleep quality (*P*=0.033). There was no observed significant difference in sleep quality score based on gender, age groups, number of active SIM cards, educational terms and level, residency place and marital status (Table 3).

**Table 2:** Comparison the sleep quality score in studied students based on social network of mobile

**Social networks**	**Not Used**		**Used**		***P*** ** value**
	**Mean**	**SD**	**Mean**	**SD**	
Viber	4.98	2.18	5.54	2.43	0.026
WhatsApp	4.98	2.39	5.52	2.29	0.032
Tango	5.12	2.26	6.05	2.56	0.002
Line	5.07	2.29	5.74	2.39	0.008
Instagram	5.23	2.38	5.51	2.48	0.323
Others	5.33	2.33	5.19	2.43	0.672

**Table 3:** Comparison the sleep quality score in studied students based on demographic characteristics

**Variables**	**Sleep Quality Score**		***P*** ** value**
	**Mean**	**SD**	
Gender			0.182
Female	5.42	2.35	
Male	5.06	2.33	
Educational level			0.170
Bachelor	5.19	2.40	
General physician	5.37	2.20	
Marital status			0.638
Single	5.37	2.37	
Married	5.14	2.15	
Residency			0.033
Parent’s house	5.02	2.21	
Dormitories	5.56	2.49	
Age group (yr)			0.923
<19	5.26	1.99	
20-21	5.36	2.31	
22-23	5.15	2.52	
≥24	5.28	2.31	
Number of SIM card			0.127
1	5.15	2.24	
≥2	5.55	2.52	
Semester			0.795
≤3	5.35	2.25	
4-6	5.14	2.44	
≥7	5.17	2.30	

The mean score of sleep quality and different components of sleep quality is showed in Table 4. Sleep disturbances score was the poorest components of sleep quality and habitual sleep efficiency component was the best. However, the mean of all components of sleep quality and the whole score of sleep quality showed a direct significant correlation (r=0.369, P<0.001) by cell-phone addiction score. Nevertheless, the correlation of sleep duration score was negative by cell-phone addiction score. Moreover, there was a significant difference between normal and over-used cell-phone subjects regarding subjective sleep quality, sleep disturbances, use of sleeping medication, daytime dysfunction and total sleep quality. Nevertheless, this difference was not observed in sleep latency, sleep duration and habitual sleep efficiency.

**Table 4:** The sleep quality score and their different components in normal and over-used students

**Components**	**Overall Score**		**Normal**		**Over-used**		***P*** ** value**
	**Mean**	**SD**	**Mean**	**SD**	**Mean**	**SD**	
Subjective sleep quality score	0.99	0.75	0.93	0.71	1.50	0.88	0.001
Sleep latency Score	1.05	0.81	1.04	0.79	1.17	0.91	0.310
Sleep duration score	1.00	0.79	1.01	0.78	0.90	0.88	0.380
Habitual sleep efficiency score	0.06	0.34	0.05	0.30	0.15	0.54	0.257
Sleep disturbances score	1.70	0.53	1.03	0.52	1.33	0.53	0.002
Use of sleeping medication score	0.16	0.51	0.13	0.48	0.33	0.70	0.025
Daytime dysfunction score	0.97	0.89	0.90	0.87	1.48	0.91	0.001
Sleep quality Score	5.30	2.35	5.10	2.24	6.90	2.60	0.001

Multivariate analysis (Table 5) showed that affected to cell-phone addiction, male gender and studying in general practitioner (GP) level are the most important predictors of poor sleep quality in medical students. So that, cell-phone addiction increased the probability of poor sleep quality more than 4.5 fold (OR=4.52, CI 95%: 1.8, 1.9). In addition, male students were at risk for 50% higher for poor sleep quality (OR=1.54, CI 95%: 1.08, 2.43) and studying in GP level was related with 61% increase in poor sleep quality (OR=1.61, CI 95%: 1.18, 2.54).

**Table 5:** The logistic regression results of poor sleep quality in medical students

**Variables**	**Normal sleep quality**	**Poor sleep quality**	**Odds ratio (95% CI)**	***P*** ** value**
Cell-phone addiction				
No	134	190	1.00	
Yes	5	34	4.52 (1.80, 11.90)	0.002
Gender				
Female	90	161	1.00	
Male	49	63	1.54 (1.08, 2.43)	0.048
Educational level				
Bachelor	99	137	1.00	
General physician	39	86	1.61 (1.18, 2.54)	0.040

Multivariate model adjusted based on the related covariates in univariate analysis including educational level, residency place, SIM card number and using social networks.

## Discussion

According to results, male students studying in GP level are at risk of higher usage of mobile cell-phones and more addiction to cell-phones due to more contribution in social networks. Therefore, cell-phone addiction due to higher usage of social networks is effective on sleep quality and quantity in medical students. However, the relationship between over-use of cell-phones, internet addiction and sleep quality is reported^16,24-26^,. University as one of the new places for students can cause sleep disturbance and decrease the sleep quality^6^. Another study in Turkish students proved the strong relationship between internet addiction and sleep^8^. Moreover, In Peru, suffering sleep disorders in people affected to Facebook addiction was 30% higher than normal people^7^. However, nowadays by increasing the touch and Android phones, the usage of web social networks increased via mobile cell-phones. Moreover, installing social networks applications on the android cell-phones enhanced the time wasting in the virtual webs and social networks. Therefore, internet addiction and cell-phone addiction are related due to both web and application’s social networks ^8,18,27^.

According to univariate results, more usage from common social networks such as Viber, WatsApp, Line, and Tango observed in students with overused smartphones. Most of the students reported using these applications in the last hours of night or after midnight instead of having rest. These nightly engagements disturbance the sleep and wake cycle due to effect on melatonin spatter and increase the risk of mental health problems such as depression, stress, anxiety, and social dysfunction^10,16,28^. Melatonin is a hormone made by the Pineal gland; a small gland in the brain increased during the darkness and the highest natural levels of blood melatonin is in the night^29^. In addition, serotonin is responsible for happiness and freshness in day times^30^. Therefore, people who are busy with smartphone all the night and prefer to sleep in the daytime are affected to stress, anxiety and depression due to melatonin and serotonin deficiency and their brain and cognitive functions would be declined^9,29,31^. Since the brain can process information and prepare for actions during sleep by keeping information and delete those are not important^31^, lack of sleep can cause problems in brain process and receiving the information from environment. The current study results also showed the higher daily dysfunction in students affected to over-used cell-phone.

Male students and who studied in GP level were more addicted to smart cell-phones and suffered from poor sleep quality. In Iran, male students that used more chat rooms and resident in dormitories were more addicted to internet^14^. The same results acquired in current study. Moreover, half of studied subjects were students resident in university dormitories and deprived from family or relatives and entertainments with their friends such as picnic or party. Therefore, the student spend his/her time to be online in his smartphone instead of having useful social activity, useful entertainment, and educational activity. Unclear future is a depression risk factor for students^32^. However, lack of opportunity and facilities for social and entertaining activities are the reasons of using the smart cell-phone and social networks and this caused the poor sleep quality. It is clear that cheap and easy access of cell-phone entertainments help to increase the chance of sleep disorder in students. Today the researchers are focusing on sleep disorder that is one of the internet addiction complications via smartphones. Sleep disorder as one of the most important of internet addiction complication is related to headache, learning disability, memory diagnosis, aggressive behavior, and mental disorder and higher risk is cause of heart disease and diabetes^8,9,16,17,26,33^. However, future studies with multicenter design are suggested with higher sample size to assess the cultural and economic differences.

## Conclusions

High usage of internet and social networks via smart cell-phones is related to poor sleep quality and quantity. Predefined sport, educational, cultural and interesting entertainment are the essential needs for all medical students especially in male students who are longer courses and are away of families to preventing cell-phone addiction and lower usage from social networks in smartphones.

## Conflict of interest statement

The authors have no conflict of interest to declare.
